# Integrated analysis of miRNA and mRNA expression profiles in tilapia gonads at an early stage of sex differentiation

**DOI:** 10.1186/s12864-016-2636-z

**Published:** 2016-05-04

**Authors:** Wenjing Tao, Lina Sun, Hongjuan Shi, Yunying Cheng, Dongneng Jiang, Beide Fu, Matthew A. Conte, William J. Gammerdinger, Thomas D. Kocher, Deshou Wang

**Affiliations:** Key Laboratory of Freshwater Fish Reproduction and Development (Ministry of Education), Key Laboratory of Aquatic Science of Chongqing, School of Life Sciences, Southwest University, Chongqing, 400715 P. R. China; State Key Laboratory of Freshwater Ecology and Biotechnology, Institute of Hydrobiology, Chinese Academy of Sciences, Wuhan, P. R. China; Department of Biology, University of Maryland, College Park, MD USA

**Keywords:** miRNA, mRNA, Tilapia, Early sex differentiation

## Abstract

**Background:**

MicroRNAs (miRNAs) represent a second regulatory network that has important effects on gene expression and protein translation during biological process. However, the possible role of miRNAs in the early stages of fish sex differentiation is not well understood. In this study, we carried an integrated analysis of miRNA and mRNA expression profiles to explore their possibly regulatory patterns at the critical stage of sex differentiation in tilapia.

**Results:**

We identified 279 pre-miRNA genes in tilapia genome, which were highly conserved in other fish species. Based on small RNA library sequencing, we identified 635 mature miRNAs in tilapia gonads, in which 62 and 49 miRNAs showed higher expression in XX and XY gonads, respectively. The predicted targets of these sex-biased miRNAs (e.g., miR-9, miR-21, miR-30a, miR-96, miR-200b, miR-212 and miR-7977) included genes encoding key enzymes in steroidogenic pathways (*Cyp11a1*, *Hsd3b*, *Cyp19a1a*, *Hsd11b*) and key molecules involved in vertebrate sex differentiation (*Foxl2*, *Amh*, *Star1*, *Sf1*, *Dmrt1*, and *Gsdf*). These genes also showed sex-biased expression in tilapia gonads at 5 dah. Some miRNAs (e.g., miR-96 and miR-737) targeted multiple genes involved in steroid synthesis, suggesting a complex miRNA regulatory network during early sex differentiation in this fish.

**Conclusions:**

The sequence and expression patterns of most miRNAs in tilapia are conserved in fishes, indicating the basic functions of vertebrate miRNAs might share a common evolutionary origin. This comprehensive analysis of miRNA and mRNA at the early stage of molecular sex differentiation in tilapia XX and XY gonads lead to the discovery of differentially expressed miRNAs and their putative targets, which will facilitate studies of the regulatory network of molecular sex determination and differentiation in fishes.

**Electronic supplementary material:**

The online version of this article (doi:10.1186/s12864-016-2636-z) contains supplementary material, which is available to authorized users.

## Background

MicroRNAs (miRNAs), a class of small non-coding RNAs, have emerged as an important and abundant group of gene expression regulators in a wide range of species [1]. Mature miRNA sequences are approximately 20–24 nucleotides (nt) long, and are formed from longer primary miRNAs that contain hairpin stem-loop structures [2]. miRNAs recognize the complementary 3’-untranslated regions (3’-UTRs) of mRNAs to inhibit their expression [3–6]. Each miRNA may have multiple gene targets, and each gene target may also be regulated by multiple miRNAs [7, 8]. miRNAs are often expressed in a tissue-specific manner, and a large proportion of protein coding genes of animals are regulated by miRNAs [9]. miRNAs are key molecules in many biological processes, including apoptosis, cell proliferation and tissue development.

Among teleosts, miRNAs were first examined in zebrafish [10]. miRNA catalogs of various tissues in other fish species were characterized more recently [11–17]. Currently, 1623 miRNAs have been identified in 9 teleost species, compared to 2588 miRNAs in human (miRBase 21.0, http://www.mirbase.org/) [18], suggesting that more miRNAs await characterization in fishes. Next generation sequencing facilitates the profiling of both known and novel miRNAs, especially those expressed in low abundance. Data from such deep sequencing will help to characterize the structure of miRNA molecules, and will provide quantitative measurement of their expression.

Teleosts are the largest group of vertebrates and exhibit a remarkable variety of sexual systems [19, 20]. Genes, environmental factors, and even social interaction may contribute to sex determination and differentiation in a large number of fish species [21]. Compared to other vertebrates, the molecular mechanisms of sex determination and differentiation in teleosts are quite diverse [22]. Over the past few years, seven master sex-determining genes have been identified in teleosts, namely *dmy*/*dmrt1b*^*Y*^ in *Oryzias latipes* and *O. curvinotus* [23, 24], *gsdf*^*Y*^ in *O. luzonensis* [25], *sox3* in *O. dancena* [26], *amhy* in *Odontesthes hatchery* [27] and *Oreochromis niloticus* [28], *amhrII* in *Takifugu rubripes* [29], *gdf6Y* in *Nothobranchius furzeri* [30] and *sd*^Y^ in *Oncorhynchus mykiss* and several other salmonids [31, 32]. However, it is well-known that sex steroid hormones, especially estrogens, may influence early sex differentiation in fishes, in spite of variation in the master sex determining genes and the non-conserved subsequent genetic steps of sex differentiation [33]. Previous studies have been focused mainly on the function of *Cyp19a1a*, which encodes the key enzyme responsible for the conversion of androgens to estrogens [34–36]. The expression of *Cyp19a1a* in the gonads is mediated by both *Foxl2* [37] and *Dmrt1* [38, 39].

Several studies have explored the role of miRNAs in regulating sex differentiation. let-7 and miR-21 have been shown to regulate development of rainbow trout eggs [16]. miRNAs are also involved in oocyte development, hydration, and competence, indicating their importance in the regulation of oogenesis [40]. miR-141 and miR-429 have crucial functions in yellow catfish testis development and spermatogenesis [41]. Different expression pattern of miRNAs is observed between the embryos of females and males [42], as well as the ovaries and testes in tilapia gonads at later stages [17, 43], indicating their regulatory roles during fish reproduction. let-7a, miR-143, and miR-202 are upregulated to induce testes differentiation in halibut [44]. Therefore, miRNAs may represent novel regulators of gonadal development and sexual differentiation. However, in these studies the small RNA libraries were generated from whole embryos or from gonads at later developmental stages, which may mask important sex-biased miRNAs in gonads at the critical stage of sex differentiation. Importantly, the regulatory roles of miRNAs during early sex differentiation of fishes have not been investigated extensively. Functional prediction of miRNAs solely based on computational miRNA-mRNA interactions bears a high number of false positive predictions [45]. Thus, the joint investigation of miRNA and mRNA expression may help us to improve the quality of predicted interactions and understand the molecular mechanisms of post-transcriptional regulation.

Nile tilapia (*O. niloticus*) is one of the most important farmed fish with a production exceeding 4.5 million tons in 2014 (http://faostat3.fao.org/home/E). The growth rate of males is significantly higher than that of females in tilapia. All-male stocks are preferred because they improve the efficiency of commercial production. It is therefore important to understand the molecular mechanism of sex determination in this species. Thus, quantifying the expression of both miRNAs and mRNAs in the undifferentiated gonads at the critical stage of molecular sex determination [46], approximately 5 days after hatching (dah), might help to clarify the regulatory network during early sex differentiation and provide new information on the role of miRNAs in gonadal function.

## Results

### Identification of conserved miRNAs in the tilapia genome

**Fig. 1:**
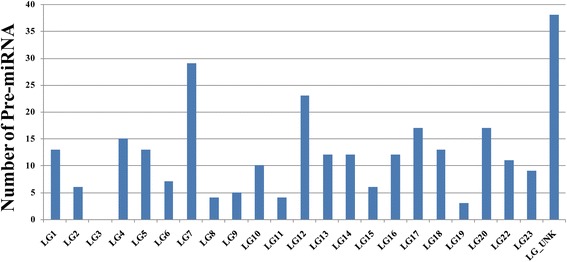
Genomic distribution of identified pre-miRNAs in tilapia

**Fig. 2:**
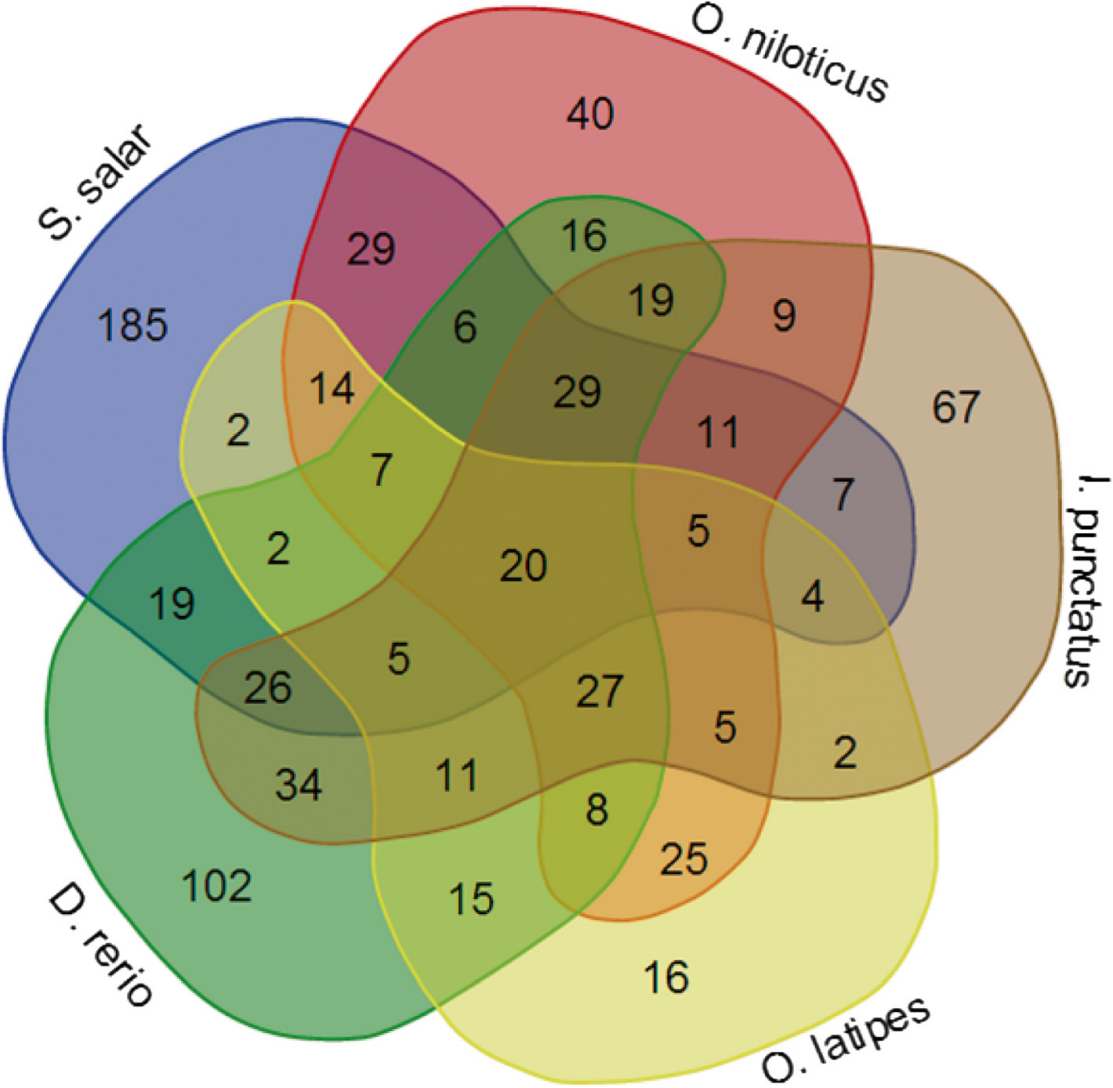
Comparisons of the number of pre-miRNAs of *Oryzias latipes, Ictalurus punctatus, Danio rerio and Salmo salar* in miRbase with the number of pre-miRNAs identified in *O. niloticus*

The reference set of 1623 known teleost pre-miRNA sequences was used for a similarity search against the tilapia reference genome assembly. We manually checked the best hits to extract putative tilapia pre-miRNAs and confirmed that they were able to fold into the secondary stem-loop structure necessary for miRNA biogenesis. This resulted in the prediction of 279 distinct tilapia pre-miRNA genes (Additional file 1: Table S1). Pre-miRNAs were non-randomly distributed in the tilapia genome (*X*^2^, *p* < 0.05). No pre-miRNAs were observed on LG3, while 29 were observed on LG7 (Fig. 1). Also, some pre-miRNAs were encoded at a single chromosomal location (e.g. miR-100 on LG10, and miR-106a on LG1), while others were encoded on multiple chromosomes (e.g. miR-103 on LG2, LG13 and LG19; miR-124 on LG9, LG15, LG19 and LG20). Comparisons of the number of pre-miRNAs identified in the tilapia genome with those reported in other fishes were shown in Fig. 2 and Additional file 2: Figure S1. The data suggested that most of the pre-miRNAs identified in tilapia have homologs in other fishes. To further study the conservation and evolution of the known pre-miRNAs in teleosts, sequence comparisons were also made with *Astatotilapia burtoni*, *Neolamprologus brichardi*, *Pundamilia nyererei*, *Metriaclima zebra*, *Oryzias latipes*, *Gasterosteus aculeatus* and *Danio rerio*. Based on these analyses, most of the pre-miRNAs showed a high level of conservation among the various fish species (Additional file 3: Figure S2).

### High-throughput sequencing of miRNAs in tilapia gonads

**Fig. 3:**
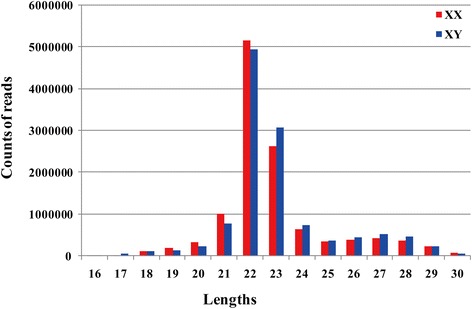
Length distribution of miRNA sequences in ovary and testes of tilapia by Illumina small RNA deep sequencing. Sequence length distribution of clean reads based on the abundance and distinct sequences; the most abundant size class was 22 nt, followed by 23 nt and 21 nt

Deep sequencing of miRNAs from tilapia XX and XY gonads at 5 dah was performed using the Illumina HiSeq 2000 platform. Approximately 12 million reads were obtained from each library. After filtering low-quality reads and empty adaptor sequences, 11,065,356 sequences from the XX gonad and 11,123,822 sequences from the XY gonad were mapped to the tilapia genome, amounting to 92.6 and 91.5 % of the total miRNA libraries, respectively (Table 1). A total of 8,203,487 (XX gonads) and 8,352,968 (XY gonads) reads were mapped to miRBase entries, representing a total of 635 miRNAs (Additional file 4: Table S2). In both XX and XY gonads, the majority of miRNAs were in the range of 21 to 24 nts, accounting for 70.18 and 64.05 % of the total mappable reads (Fig. 3). The average length of the mature miRNAs in the XX and XY gonad were 22.8 and 23.0 bp, respectively.

**Table 1:** Number of high-throughput reads generated from miRNA libraries of tilapia XX and XY gonads at 5 dah

	Raw reads	Clean reads	Map to genome	Map to miRBase
XX	12,175,326	11,946,840	11,065,356 (92.6 %)	8,203,487 (68.67 %)
XY	12,378,698	12,157,106	11,123,822 (91.5 %)	8,352,968 (68.71 %)

**Fig. 4:**
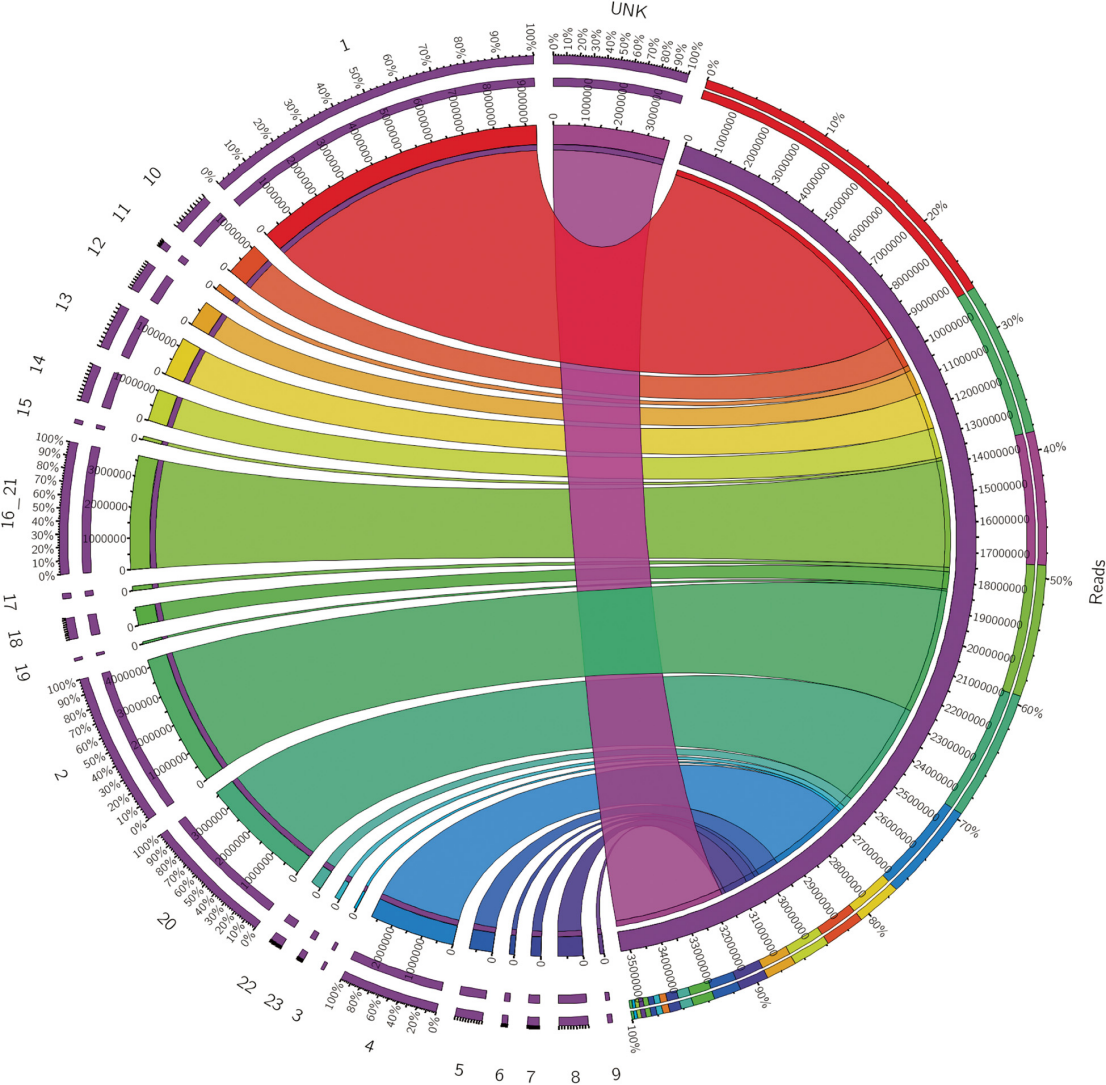
Circos circular visualization of the miRNA reads coverage on tilapia genome

To profile the chromosomal distribution of active miRNAs detected, the read counts for each chromosome from both XX and XY gonads were consolidated and the percentage of transcribed miRNA reads per chromosome was computed. Approximately 40 % of the mature miRNAs were localized to LG 1, 2, 16, and 20 (Fig. 4). Only a few reads were mapped to LG3, which is large and known to be highly repetitive. The identified miRNAs in tilapia covered a large proportion of reported miRNAs in fishes, such as *D. rerio*, *T. nigroviridis*, *T. rubripes*, *O. latipes* (Additional file 5: Figure S3). Most of the previously reported cichlid miRNAs [47] exhibited orthology to the identified miRNAs in the present study (Additional file 6: Figure S4).

The top 7 most abundant miRNAs in tilapia gonads included miR-10a-5p, miR-10b, miR-10c-5p, miR-10d, miR-21, miR-100, and miR-181a-5p and accounted for 72.89 % (74.99 %) of the 8,203,487 (8,352,968) reads mapped to miRBase. Sequence analysis indicated that the relative abundance of miRNAs from one miRNA family varied greatly in the tilapia gonads, suggesting possible functional divergence among different members within the miRNA family. For example, abundance of different members of the miR-10 family varied from 9 reads (miR-10c-3p) to 2,765,827 and 2,848,186 (miR-10b) in tilapia XX and XY gonads, respectively.

### Identification of novel sex-biased miRNAs in tilapia gonads

From the reads mapped to tilapia genome, we identified potentially novel miRNAs that were not matched to any sequences in the miRBase. Based on analyzing the surrounding sequences (50 nts on both directions) for the ability to form hairpin structures, 130 novel miRNAs displayed sex-biased expression (Additional file 7: Table S3). These putative miRNAs showed sequences variations at the 5’ or 3’ terminus compared with known miRNAs in miRBase. Of the 130 novel miRNAs, 49 and 45 miRNAs were identified uniquely in XX and XY gonads, respectively. A greater proportion of the novel miRNAs were sex-biased than were the set of known miRNAs.

### Identification of miRNAs uniquely expressed in tilapia XX and XY gonads

Among the 635 mature miRNAs identified in the two libraries, 557 miRNAs were expressed in both libraries. We next directed our attention to the miRNAs that were identified exclusively from either XX or XY gonads. 39 miRNAs including miR-34c-5p, miR-153-5p, miR-749, and miR-2187-5p were expressed exclusively in XX gonads, while 49 miRNAs including miR-1306-5p, miR-132b and miR-18c were expressed exclusively in XY gonads. However, no miRNAs appeared exclusively in either sex with more than 15 reads.

### Identification of miRNAs differentially expressed in tilapia gonads

**Fig. 5:**
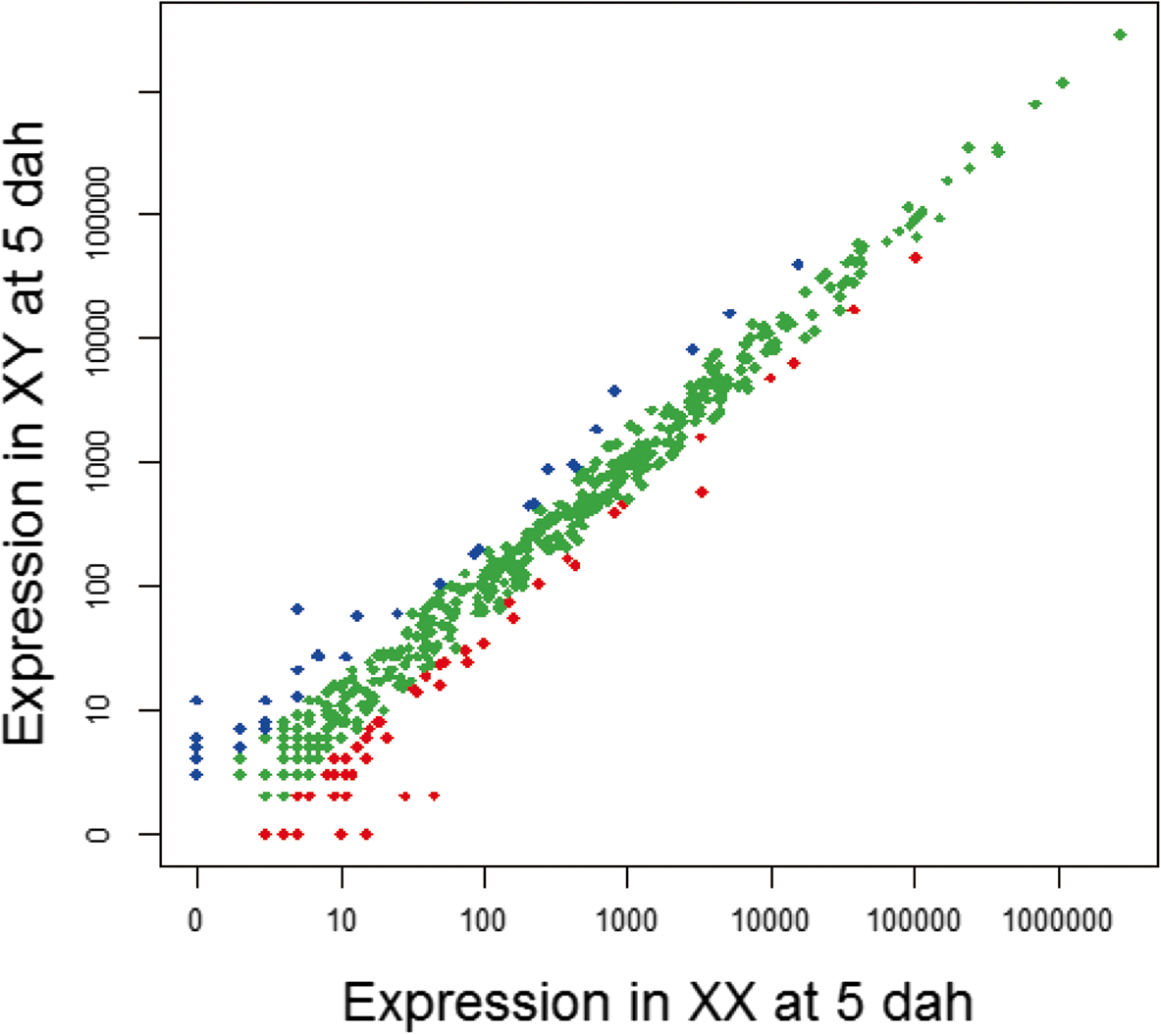
Comparison of expression levels of miRNAs in XX and XY gonads. The X axis and Y axis show expression level of miRNAs in the two samples, respectively. Red points represent miRNAs with ratio > 2; green points represent miRNAs with 1/2 < ratio ≤ 2; blue points represent miRNAs with ratio ≤ 1/2. Ratio = normalized expression of XX gonad/normalized expression of XY gonad

We then compared the transcription level of differentially expressed miRNAs between XX and XY libraries to define miRNAs related to early ovary or testis differentiation. 62 miRNAs showed higher expression levels in XX gonads, whereas 49 were more highly expressed in XY gonads (Fig. 5). miR-9, miR-101, miR-192, miR-202, miR-212, miR-217a, and miR-737 showed greater expression in ovaries. The miRNAs upregulated in XY gonads included miR-27d, miR-29b, miR-92b, miR-140-3p, miR-144-5p, miR-375, miR-455 and miR-7977. Some abundantly expressed miRNAs also showed sex-biased expression with ~1.5 fold change, including miR-21, miR-96, and miR-200b.

### Identification of miRNAs involved in steroidogenic pathways and early sex differentiation

A total of 10,740 target genes were predicted for the 635 miRNAs by miRanda and Targetscan. The predicted modes of action for the predominant miRNAs in gonad included: 1) a single miRNA targeting a single or multiple position(s) in the 3’ UTR; 2) multiple miRNAs targeting different positions in the 3’ UTR of the same gene. The expression of target genes was quantified by mRNA sequencing at 5 dah. Sequencing of the mRNA libraries yielded ~800 million reads for female and male pool. In order to identify major miRNAs with possible role in early sex differentiation, we looked for target genes which showed inverse expression patterns compared to female and male upregulated miRNAs (Additional file 8: Table S4 and Additional file 9: Table S5). On average, miRanda and Targetscan predictions suggested about 4000 targets per miRNA compared to integrative analysis of miRNA and mRNA expression data which suggested about 90 target predictions per miRNA.

**Fig. 6:**
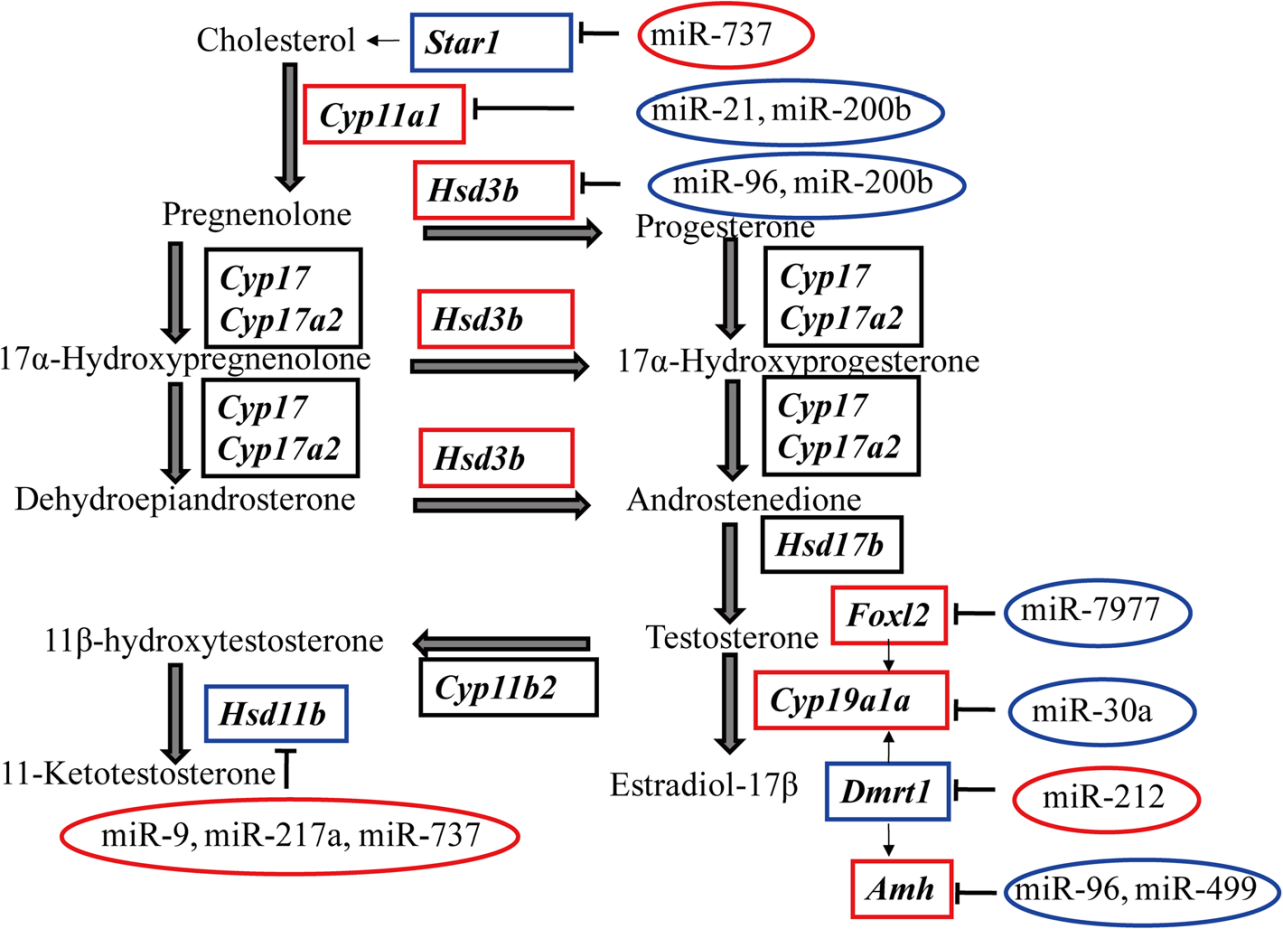
miRNA-gene network in the steroid hormone biosynthesis pathway [48] and regulatory network in early sex differentiation of Nile tilapia. The genes in boxes are differentially expressed in the mRNA-Seq and were validated by previous transcriptomic analyses [72]. Blue box nodes represent upregulated genes in XY gonads, red box nodes represent upregulated genes in XX gonads, and black box nodes represent genes with no significant difference between XX and XY. miRNAs in the ellipses potentially regulated the corresponding gene expression. Blue ellipses represent downregulated miRNAs in XX gonads; red ellipses represent downregulated miRNAs in XY gonads

Subsequently, we examined the expression of key genes known to be involved in the steroid hormone biosynthesis pathway [48] and those that participate in vertebrate sex differentiation [46]. *Cyp19a1a*, *Foxl2*, *Cyp11a1*, *Hsd3b* and *Amh* showed female upregulated expression, while *Dmrt1* and *Hsd11b* showed up-regulation in males at 5 dah. Nine microRNAs (miR-30a, miR-30a-3p, miR-30b-3p, miR-30d-3p, miR-30e-3p, miR-153--5p, miR-205b-3p, miR-737-5p, and miR-737-3p) were predicted to regulate the expression of *Cyp19a1a*. Among these 9 microRNAs, all members of miR-30 family showed down-regulation in females, though the difference was not a 2-fold decrease. *Dmrt1* was predicted to be the target of miR-9-3p, miR-22-5p, miR-132, miR-212, miR-212-3p and miR-7641, and the expression of miR-212-3p was downregulated in males. miR-203a, 203-3p, miR-218, miR-429, miR-722, and miR-7977 were predicted to target *Foxl2*, and the expression of miR-7977 was downregulated in the XX gonad (Fig. 6 and Additional file 10: Figure S5). Interestingly, some miRNAs targeted two or more genes in the regulatory network of tilapia gonadal differentiation: miR-96 targeted both *Amh* and *Hsd3b*, while miR-737 targeted both *Star1* and *Hsd11b*. Other miRNAs targeting key genes in the pathway for sex steroids synthesis and early sex differentiation, including *Gsdf* and *Star1*, were listed in Additional file 11: Table S6.

## Discussion

miRNAs regulate a vast number of genes [9], which makes them likely to play a critical role in programming the ovary and testis. In the present study, both *in silico* prediction of conserved pre-miRNAs and an integrated analysis of deep sequencing of miRNA and mRNA from XX and XY gonads at the critical stage of sex determination were performed to identify conserved miRNAs in fish and sex-associated miRNAs in tilapia.

Previous bioinformatic studies identified 21 conserved miRNAs and 100 pre-miRNAs in cichlid fishes, using EST, GSS and partial genomic sequences [49, 50]. The present study identified a total of 279 conserved pre-miRNAs, by comparing the published tilapia genome to pre-miRNAs deposited in miRBase 21.0. The increase can be attributed to the submission of additional fish miRNAs to miRBase, and to an improved assembly of the tilapia genome.

The number of miRNA precursors varied significantly among chromosomes. The smallest number of miRNAs was identified on LG3 by both *in silico* prediction and miRNA reads mapping, even though it is the longest chromosome in the karyotype. LG7 carries a higher number of miRNA genes, suggesting an important role for this chromosome in regulating tilapia tissue development and differentiation as in human [51]. miRNAs are also reported to be non-randomly distributed in the human and mouse genomes [52–54]. In addition, the multiple locations of the same miRNA on different LGs, such as pre-miR-103 and pre-miR-124, suggest the possibility that transcription of the same miRNA from different sites may regulate the expression of target genes in different tissues or at different development stages [55].

In fish, the miRNA distribution in organs of the reproductive axis (brain, pituitary and gonad) has recently been reported in mature stages of the Atlantic halibut [44], yellow catfish [41], and Nile tilapia [17, 43]. However, to date there is little data on miRNAs that have a possible role in early sex differentiation, especially in fish gonads. Only one previous study identified 9 sexually differentially expressed miRNAs in early developing tilapia embryos [42]. Thus, we performed miRNA and mRNA Illumina sequencing on the same gonadal sample to identify the complete set of known and novel miRNAs, as well as explore their possible roles in early sex differentiation.

The miRNA libraries of XX and XY gonads displayed similar read length distribution after filtering high quality sequencing. The most abundant miRNAs in both libraries were 21 and 22 nt, followed by 23 nt, representing the typical size of Dicer-derived products, and is consistent with the length distribution of miRNA in mouse [56]. Ovary and testis at the mature stages in zebrafish and tilapia show significant piRNA expression with another peak at 26–28 nt due to the abundant expression of PIWI-interacting RNAs (piRNAs) [17, 57]. In the present study, there were more miRNAs with length of 21 and 22 nt in XX gonads, whereas in XY gonads there were more miRNAs with length of 23 nt, which has also been observed in a previous study of gonadal miRNAs in tilapia [17].

In tilapia gonads at 5 dah, the most abundant miRNAs were miR-10a-5p, miR-10b, miR-10c-5p, miR-10d, miR-21, miR-100, and miR-181a-5p, each with more than several thousand reads. As in human, members of miR-10 family dominate the miRNA population in both ovary and testis, consistent with a relationship of members of miR-10 with sex hormones [58]. Expression of miR-21 increases substantially following the induction of differentiation, suggesting its important roles in stem cell differentiation [59] and endocrine regulation [42, 60]. miR-100 is also highly expressed in mature ovaries and testes of yellow catfish [41], indicating its possible involvement in gonadal development. The miR-181a family is abundantly expressed in the gonads of tilapia [17], mouse [61], and human [62], which suggests that this family also may play a crucial role in sex determination and differentiation. The most abundantly expressed miRNAs in tilapia XX and XY gonads at 5 dah are functionally conserved with other vertebrates, indicating that these miRNAs may play conserved role in gonadal development and differentiation. However, we also identified 130 novel sex-biased miRNAs that were not similar to any known conserved miRNAs in miRBase. These novel miRNAs with overall low abundance may be newly evolved in tilapia, or may have been overlooked in other species. The preferential expression of one of the duplexes (−5p or -3p) of mature miRNA (e.g. miR-9b-5p, miR-9b-3p) has also been reported from different tissues of mouse [63]. These results indicate that different duplexes within one miRNA can have clearly different expression levels, mostly due to tissue or developmental stage-specific expression.

No sex-specific miRNAs with reads higher than 15 were identified in tilapia gonads, a result that is consistent with the previous study where the expression levels of the tissue-specific expressed miRNAs were low in Holstein cattle (1–23 reads) [64]. More miRNAs were significantly upregulated in ovaries, which is also observed in adult Nile tilapia [17], medaka [65] and Holstein cattle [64]. Among these upregulated miRNAs, some have been reported to be very important in ovarian development in various species, such as miR-101 in regulating ovary differentiation in embryonic chicken gonads [66], miR-212 in ovarian development with the putative target *Wt1* and *Gata2* [67, 68]. Our results (higher expression of miRNAs in XY gonads) are in agreement with previous studies. For example, miR-140-3p is also abundantly expressed in Sertoli cells of the developing mouse testis [69]. miR-375 has been suggested to play an important role in testicular differentiation in *Xenopus* [70]. As in tilapia, miR-499 is also a dominant miRNA in swine, indicating its involvement in testicular development [71]. miR-9 and miR-192 are also upregulated in tilapia ovary, while miR-27d, miR-29b, miR-92b, miR-144-5p and miR-455 show the same pattern of greater expression in testes at later stage [43], suggesting they might play crucial roles in tilapia sex differentiation. However, the function of most other sex-biased miRNAs was previously unknown. More studies are needed to uncover the regulatory roles of these miRNAs.

By applying integrated miRNA and mRNA analysis together with target prediction, we reduced the complexity of predicted miRNA-mRNA relations on average by more than 50-fold compared to pure *in silico* prediction. Thus, we were able to explore the functional details of sex-biased miRNAs in early sex differentiation with the combined data. The transcriptomic data collected in the current work and a previous study [72] further confirmed the critical role of estrogen in sex determination and differentiation. The differentially expressed miRNAs targeted multiple genes, including those genes encoding key enzymes in the steroid hormone biosynthesis pathway (*Cyp11a1*, *Hsd3b*, *Cyp19a1a*, *Hsd11b*) [48] and key factors involved in early sex differentiation (*Foxl2*, *Amh*, *Star1*, *Sf1*, *Dmrt1*, and *Gsdf*) [46]. *Cyp19a1a* encodes the key enzyme responsible for estrogen synthesis. XX gonads with higher expression of *Cyp19a1a* exhibited lower expression of miR-30, which suggests a critical role of the miR-30 family in early sex differentiation via ovarian cell steroidogenesis as in human [73]. However, how the ovary is differentiated through *Cyp19a1a* expression being regulated by the miR-30 family remains to be investigated. Furthermore, *Foxl2* and *Dmrt1* play antagonistic roles in sex differentiation in Nile tilapia via regulation of *Cyp19a1a* expression and estrogen production [39]. Elevated expression of *Foxl2* and decreased expression of miR-7977 in tilapia XX gonads indicates the possible role of miR-7977 in early sex differentiation. The higher expression of miR-212, which may repress the expression of *Dmrt1* in the XX gonad, suggests its possible role as a post-transcriptional regulator in the differentiation of granulosa cells, as demonstrated in mouse [74]. Interestingly, some miRNAs (e.g., miR-96 and miR-737) target multiple genes involved in steroid synthesis, suggesting the complex regulatory network in fish sex determination and differentiation. Hence, these results suggest a role for miRNAs in regulating the biosynthesis of steroid hormones during tilapia early sex differentiation.

## Conclusions

The present study is the first to examine the miRNA and mRNA expression profile of tilapia XX and XY gonads in early sex differentiation. We identified 111 known miRNAs that were differentially expressed between the XX and XY gonads. The predicted targets of these sex-biased miRNAs included genes encoding key enzymes in steroidogenic pathways and key molecules involved in vertebrate sex differentiation. These genes also showed sex-biased expression in tilapia gonads at 5 dah based on transcriptomic analysis. Some miRNA (e.g., miR-96 and miR-737) targeted multiple genes involved in steroid synthesis, indicating the complex regulatory network in fish early sex differentiation. An additional 130 novel miRNAs with differential expression in the XX and XY gonad were also identified. These miRNAs are an interesting starting point for future research to understand miRNA-mRNA interaction in early sex differentiation of teleosts. The availability of mono-sex fish and genome editing in tilapia makes it an attractive model system for study of miRNA function during sex determination and differentiation.

## Methods

### Prediction of miRNA genes using *in silico* approaches

A database of 1623 known teleost precursor miRNA (pre-miRNA) sequences was downloaded from miRBase release 21.0 [18]. To detect miRNA genes in tilapia, we conducted a blastn similarity search of these pre-miRNAs against the tilapia genome sequences [47] with an E-value cutoff of 0.001. The blastn hits were then manually inspected and compared with their query sequences in order to extract adjacent nucleotides (~30 bp) in both directions that might form part of the pre-miRNA. RNA secondary structure of the tilapia putative miRNA sequences was predicted using Mfold [75] to ensure proper stem–loop folding. A reciprocal blastn of the tilapia pre-miRNAs against known teleost pre-miRNAs was performed to annotate these predicted pre-miRNAs and to assign orthology (Additional file 12: Figure S6). Specifically, we compared the high confidence set of tilapia pre-miRNA sequences with those of cichlids [47] and other fishes from miRBase release 21.0 [18].

### Ethics

All fish experiments were conducted in accordance with the regulations of the Guide for Care and Use of Laboratory Animals and were approved by the Committee of Laboratory Animal Experimentation at Southwest University.

### Fish materials

The founder strain of the Nile tilapia, which was first introduced from Egypt in Africa, was obtained from Prof. Nagahama (Laboratory of Reproductive Biology, National Institute for Basic Biology, Okazaki, Japan). Fishes were reared in re-circulating aerated freshwater tanks at 26 °C prior to use. The breeding of mono-sex fish (all-female or all-male progenies) facilitates sampling of the gonads before the morphological differentiation. All-XX progenies were obtained by crossing a pseudomale (XX male, producing sperm after hormonal sex reversal) with a wild type female (XX). All-XY progenies were obtained by crossing a supermale (YY) with a wild type female. XX and XY fish were dissected to obtain samples of gonads for RNA isolation at the critical stage of molecular sex determination [46] (5 dah, 150 gonads were pooled for each sex).

### RNA isolation

Total RNA was extracted from gonads of XX and XY fishes frozen in liquid nitrogen using the Trizol Reagent (Invitrogen, Carlsbad, CA) according to the manufacturer’s instruction. The extracted RNA was further treated with DNaseI (RNase-free 5U/uL) to eliminate genomic DNA contamination. The integrity and concentration of RNA was determined using both agarose gel electrophoresis and Nanodrop spectrophotometer (Nanodrop Technologies, Wilmington, Delaware USA), respectively. RNA with OD260/280 = 1.8–2.1, OD260/230 ≥ 1.7, c ≥ 150 ng/μL, and RNA integrity number (RIN) ≥ 7.0 and 28S/18S > 1.5 was stored at −80 °C for subsequent library construction and sequencing.

### Illumina paired-end cDNA library construction and sequencing (RNA-Seq)

Two small RNA libraries and two mRNA libraries were constructed from purified RNA of tilapia XX and XY gonads. sRNA-Seq (small RNA sequencing) and mRNA-Seq (mRNA sequencing) services were carried out using Illumina HiSeq 2000 platform (Illumina Inc., San Diego, CA, USA) provided by BGI-Shenzhen, China. For sRNA-Seq, small RNAs of 16–32 nt in length were isolated from the total RNA by size fractionation in a 15 % TBE urea polyacrylamide gel, small RNAs were ligated to 3’ adaptor and 5’ adaptor with T4 RNA ligase. Subsequently, PCR reaction was performed using primers complementary to the two adaptors. The amplification products were further purified on 6 % polyacrylamide TBE gel and used for sequence analysis. In brief, Oligo(dT) beads were used to isolate poly(A) mRNA from total RNA for mRNA-seq. The average insert size in an mRNA sequencing library is approximately 200 bp and both ends of the library were sequenced. The reads of sRNA-Seq and mRNA-Seq have been deposited in NCBI’s Sequence Read Archive (SRA) database with accession numbers SRS1212947 and SRS1212948.

### miRNA annotation and expression profiling

For sRNA-Seq, Trimmomatic [76] and a custom PERL script was used to remove the low-quality reads and trim the adaptor (5’-GCCTTGGCACCCGAGAATTCCA-3’). After adaptor trimming, reads of 18–32 nt in length were kept for further bioinformatics analysis. These reads were mapped to Nile tilapia genome with a tolerance of one mismatch in the seed sequence using Bowtie2 [77]. Sequences with perfect matches or one mismatch were retained for further analysis. Subsequently, by blasting against the Rfam database (12.0, http://rfam.xfam.org/), the reads mapped to the Nile tilapia genome were analyzed to discard the rRNA, tRNA, snoRNA, and ncRNA sequences. The remaining sequences were identified as the conserved miRNAs in Nile tilapia by a blast search against miRBase 21.0 allowing no more than one mismatch. The detailed mapping process was performed as previously described [17]. The orthologous miRNA matches were named according to the original gene name except the species name. The remaining reads were used for novel miRNA prediction. To further analyze the RNA secondary structures for novel miRNA prediction, 50 nts of genomic sequence flanking each side were extracted, and the secondary structures with regard to miRNA precursor gene characteristics were predicted using Mfold [75] (Additional file 13: Figure S7). miRNA read counts were normalized to tags per million (TPM) counts in order to compare miRNA expression between XX and XY gonads. The TPM was calculated as follows: normalized expression, TPM = (actual miRNA count/number of total clean read) × 1,000,000. The fold-change |log_2_Ratio| ≥ 1 and *P*-value (<0.01) were calculated to identify differentially expressed miRNAs.

### Target prediction of the sex-biased miRNAs and expression profile of mRNAs

To provide a better understanding of the roles of the sex-biased miRNAs and correlations between expression levels of miRNA and mRNA, the mRNAs potentially targeted by these miRNAs were predicted using both miRanda [78] and Targetscan [79]. The 3’ UTRs for all genes of tilapia were predicted using Transdecoder [80], and were used as the primary base-pairing region of miRNAs. The prediction was based on the degree of miRNA and target sequence complementation, as well as the free energy level of RNA-RNA duplexes. Expression of mRNAs was qualified using transcriptomic analyses. Reads from mRNA-seq were aligned to the Nile tilapia reference sequence with TopHat2 [81]. NCBI RefSeq annotations were used to guide the Cufflinks [82] assembly, and Cuffdiff was used to determine FPKM values for those gene models. The results were subsequently filtered to exclude gene models whose FPKM value was less than 0.5 in either females or males. Additionally, when comparison between FPKM of the two samples was carried out, if the FPKM value exceeded 0.5 in one sex and was zero in the other sex, it was considered an undefined bias favoring the sex as the similar criteria in previous study [83]. Female upregulated and male upregulated gene models were counted exclusively against female downregulated and male downregulated miRNAs.

### Availability of supporting data

The data sets supporting the results of this article are included within the article and its additional files.

## Additional files

Additional file 1: Table S1. Sequence of identified 279 conserved pre-miRNA genes in tilapia genome. (XLS 66 kb) Additional file 2: Figure S1. Comparisons of the number of pre-miRNAs of *Paralichthys olivaceus*, *Hippoglossus hippoglossus*, *Fugu rubripe, Tetraodon nigroviridis, Cyprinus carpio, Oryzias latipes, Ictalurus punctatus, Danio rerio and Salmo salar* in miRbase with the number of known miRNAs identified in *Oreochromis niloticus*. (TIF 486 kb) Additional file 3: Figure S2. Pre-miRNAs sequence alignments of *O. niloticus*, *Astatotilapia burtoni*, *Neolamprologus brichardi*, *Pundamilia nyererei*, *Metriaclima zebra*, *O. latipes*, *G. aculeatus* and *D. rerio*. A. pre-miR-140, B. pre-miR-499. (TIF 1897 kb) Additional file 4: Table S2. Sequence and expression profiles of known miRNAs in the ovary and testis of tilapia. (XLS 1613 kb) Additional file 5: Figure S3. Comparisons of the number of mature miRNAs in *Danio rerio*, *Fugu rubripe, Tetraodon nigroviridis,* and *Oryzias latipe* in miRbase with the number of known miRNAs identified in the gonads of *O. niloticus*. (TIF 434 kb) Additional file 6: Figure S4. Venn diagram of identified miRNAs in tilapia gonads in *O. niloticus*, *A. burtoni*, *N. brichardi*, *P. nyererei* and *M. zebra* published in previous study [47]. (TIF 369 kb) Additional file 7: Table S3. Sequence and expression profiles of novel miRNAs with in the ovary and testis of tilapia. (XLS 45 kb) Additional file 8: Table S4. Predicted targets of female upregulated miRNAs validated by mRNA sequencing. (XLS 1765 kb) Additional file 9: Table S5. Predicted targets of male upregulated miRNAs validated by mRNA sequencing. (XLS 2205 kb) Additional file 10: Figure S5. Target prediction for selected miRNA expressed in ovaries and testis in tilapia. (A) *Cyp19a1a*, (B) *Dmrt1*, and (C) *Foxl2* are predicted as potential targets of miR-30a, miR-212 and miR-7977, respectively. (TIF 1109 kb) Additional file 11: Table S6. miRNAs and mRNAs in the steroid hormone biosynthesis pathway and involved in early sex differentiation. (XLS 29 kb) Additional file 12: Figure S6. Flowchart depicting the workflow for pre-miRNA prediction. A blastn similarity search of these pre-miRNAs against the tilapia genomic sequences [47] with an E-value cutoff of 0.001. The blastn hits were then manually inspected and compared with their query sequences in order to extract adjacent nucleotides (~30 bp) in both directions that might form part of the pre-miRNA. RNA secondary structure of the cichlid putative miRNA sequences was predicted using Mfold [75] to ensure proper stem–loop folding. A reciprocal blastn of the tilapia pre-miRNAs against known teleost pre-miRNAs was performed to identify the tilapia miRNA and to assign orthology. (TIF 1123 kb) Additional file 13: Figure S7. Flowchart depicting the workflow for miRNA profiling, annotation and novel miRNA prediction. The workflow comprises of five parts namely: filter of the reads, quantification of known miRNAs, the novel miRNA prediction pipeline, target prediction, quantification of mRNA. The quantification of known miRNAs and prediction of novel miRNAs was done using Mfold, and the quantification of mRNAs was performed by Tophat2 [81] and Cufflinks [82]. (TIF 1823 kb)

## Abbreviations

*Amh*: Anti-Müllerian hormone; steroidogenic acute regulatory protein 1
*Cyp11a1*: cytochrome P450 family 11 subfamily A member 1
*Cyp19a1a*: cytochrome P450, family 19, subfamily A, polypeptide 1a
*Dmrt1*: doublesex- and mab-3-related transcription factor-1
*Foxl2*: forkhead box L2
*Gsdf*: gonadal somatic cell derived factor
*Hsd11b*: 11-Beta hydroxysteroid dehydrogenase
*Hsd3b*: 3 beta- and steroid delta-isomerase 3
*Sf1*: steroidogenic factor-1
TPM: tags per million counts
