# Supplementary figures and images for: Integrated analysis of miRNA and mRNA expression profiles in tilapia gonads at an early stage of sex differentiation
Source: BMC Genomics. 2016 May 4;17:328. doi: 10.1186/s12864-016-2636-z (PMC4855716; doi:10.1186/s12864-016-2636-z)

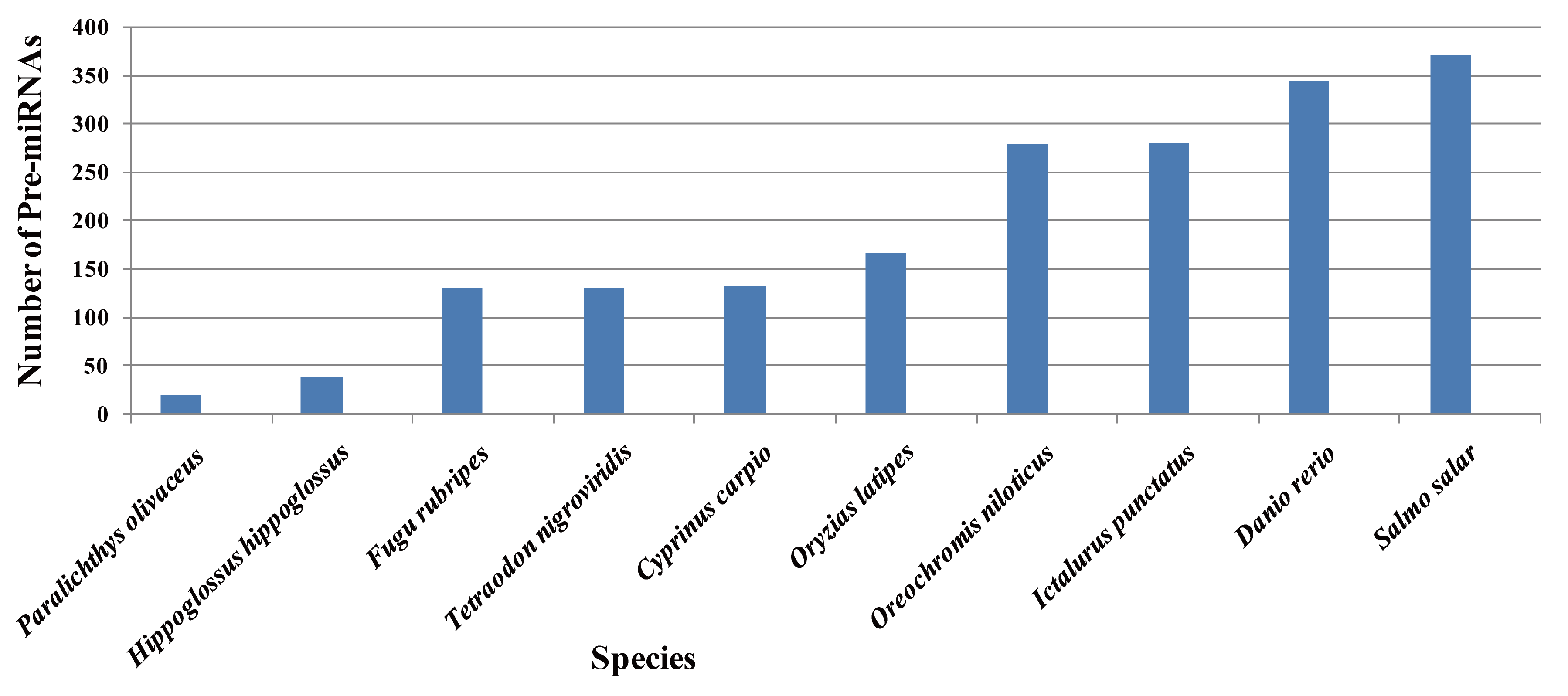

Supplement: Additional file 2: Figure S1. — Comparisons of the number of pre-miRNAs of Paralichthys olivaceus, Hippoglossus hippoglossus, Fugu rubripe, Tetraodon nigroviridis, Cyprinus carpio, Oryzias latipes, Ictalurus punctatus, Danio rerio and Salmo salar in miRbase with the number of known miRNAs identified in Oreochromis niloticus. (TIF 486 kb) [file 12864_2016_2636_MOESM2_ESM.tif]

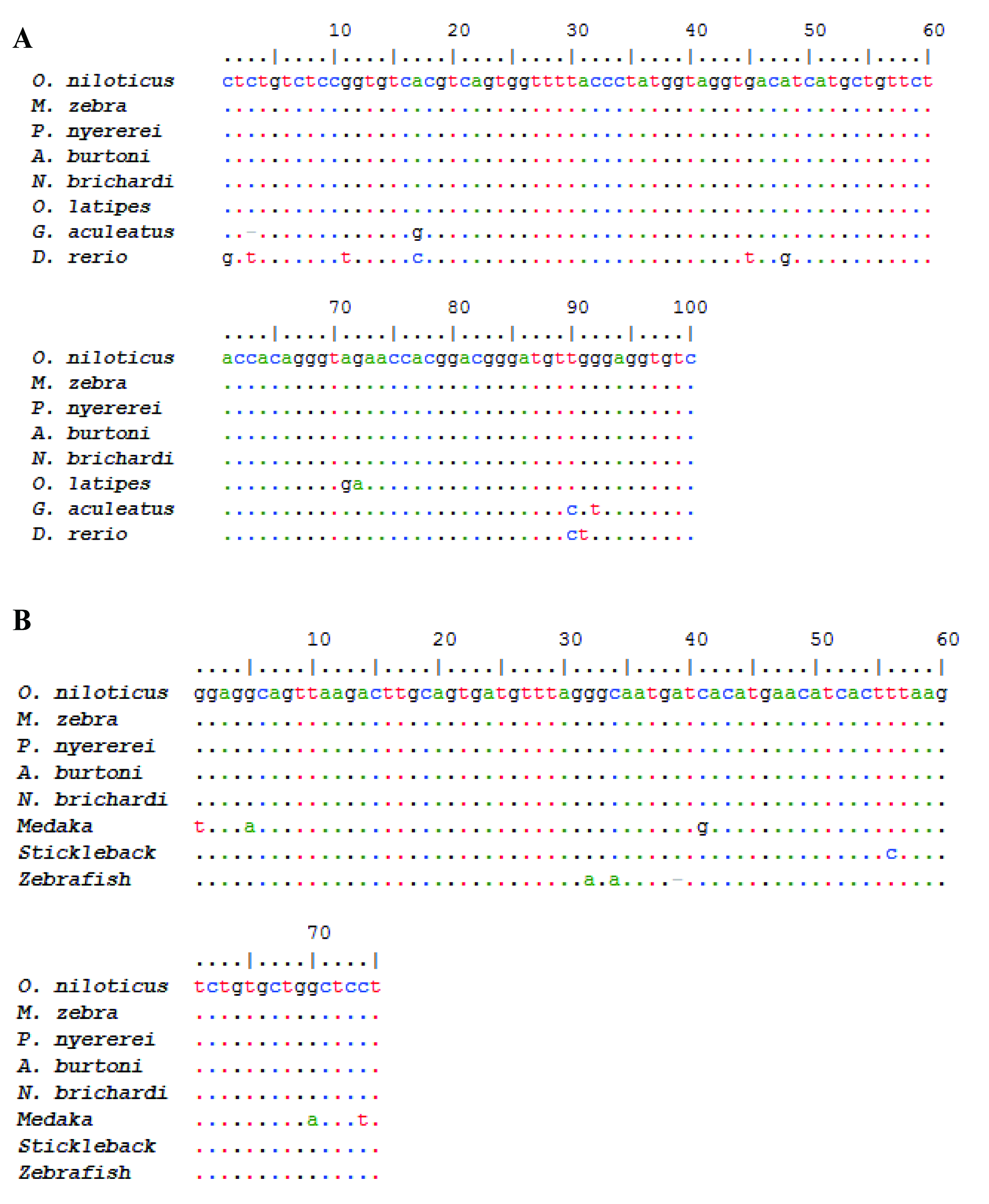

Supplement: Additional file 3: Figure S2. — Pre-miRNAs sequence alignments of O. niloticus, Astatotilapia burtoni, Neolamprologus brichardi, Pundamilia nyererei, Metriaclima zebra, O. latipes, G. aculeatus and D. rerio. A. pre-miR-140, B. pre-miR-499. (TIF 1897 kb) [file 12864_2016_2636_MOESM3_ESM.tif]

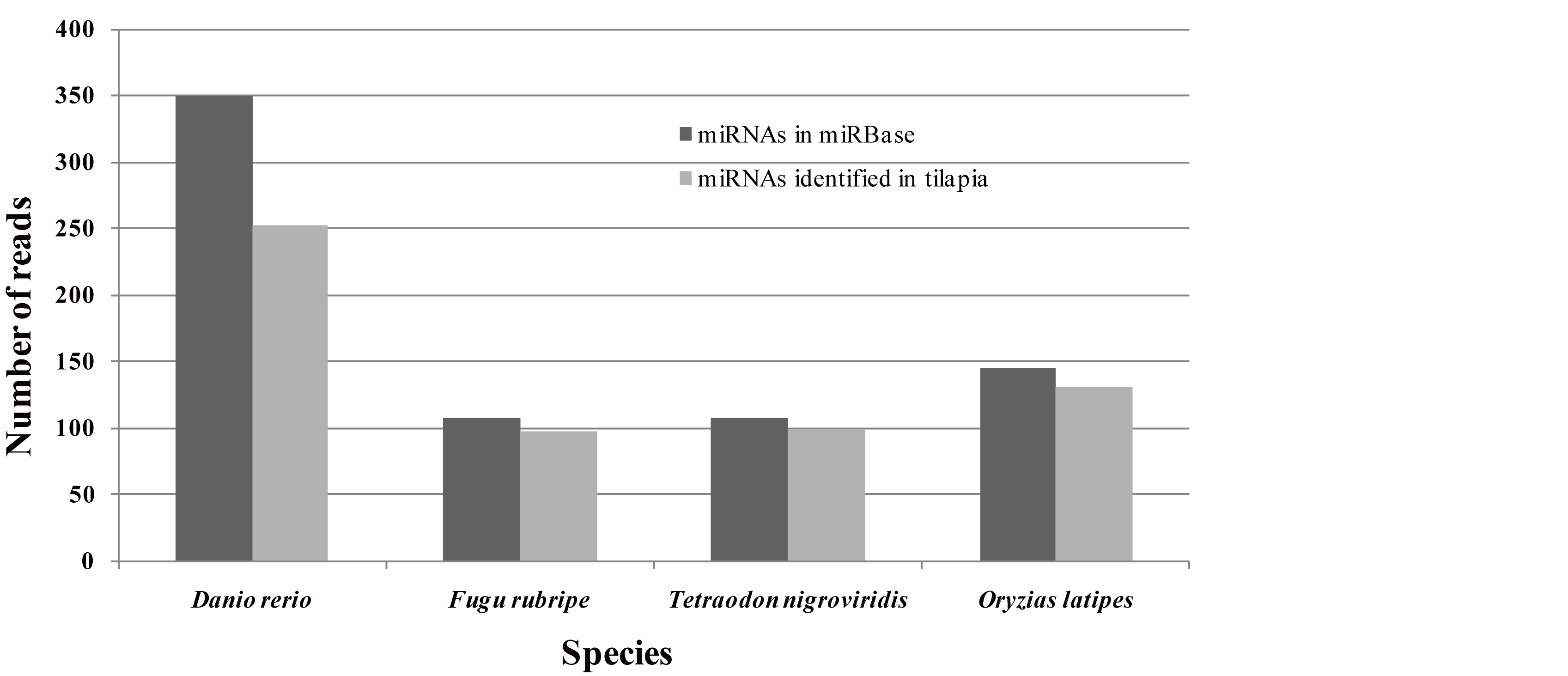

Supplement: Additional file 5: Figure S3. — Comparisons of the number of mature miRNAs in Danio rerio, Fugu rubripe, Tetraodon nigroviridis, and Oryzias latipe in miRbase with the number of known miRNAs identified in the gonads of O. niloticus. (TIF 434 kb) [file 12864_2016_2636_MOESM5_ESM.tif]

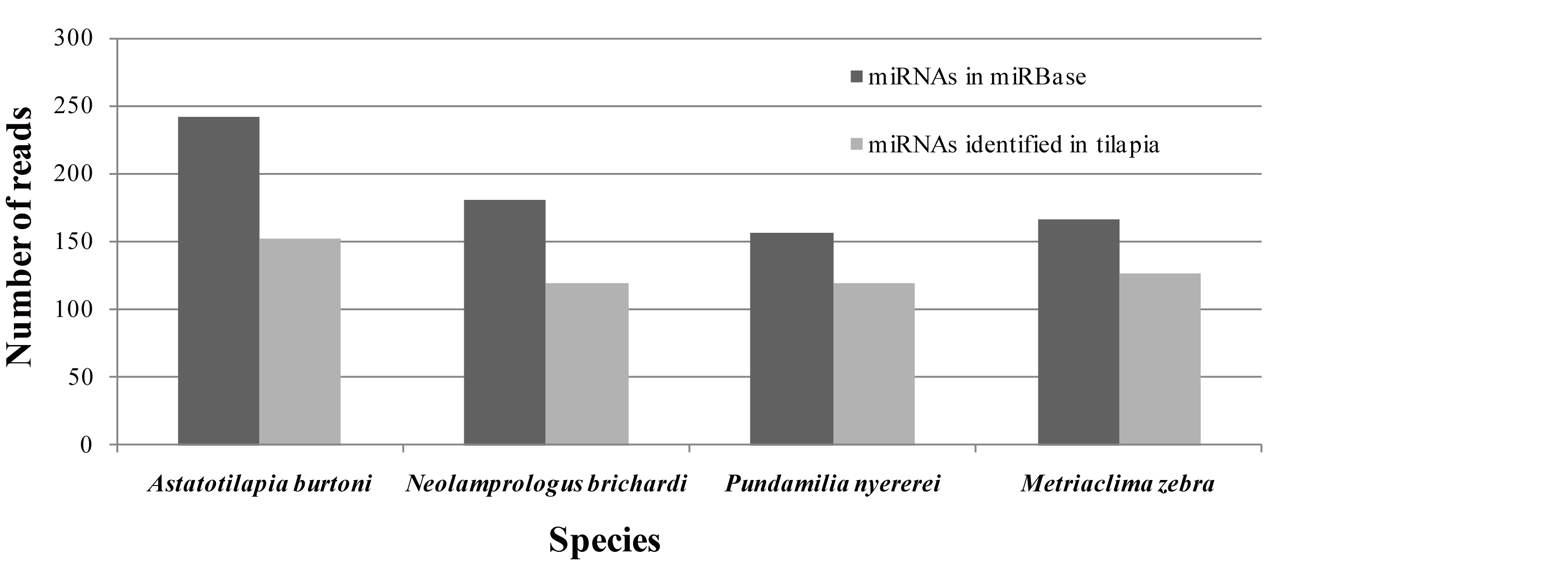

Supplement: Additional file 6: Figure S4. — Venn diagram of identified miRNAs in tilapia gonads in O. niloticus, A. burtoni, N. brichardi, P. nyererei and M. zebra published in previous study [47]. (TIF 369 kb) [file 12864_2016_2636_MOESM6_ESM.tif]

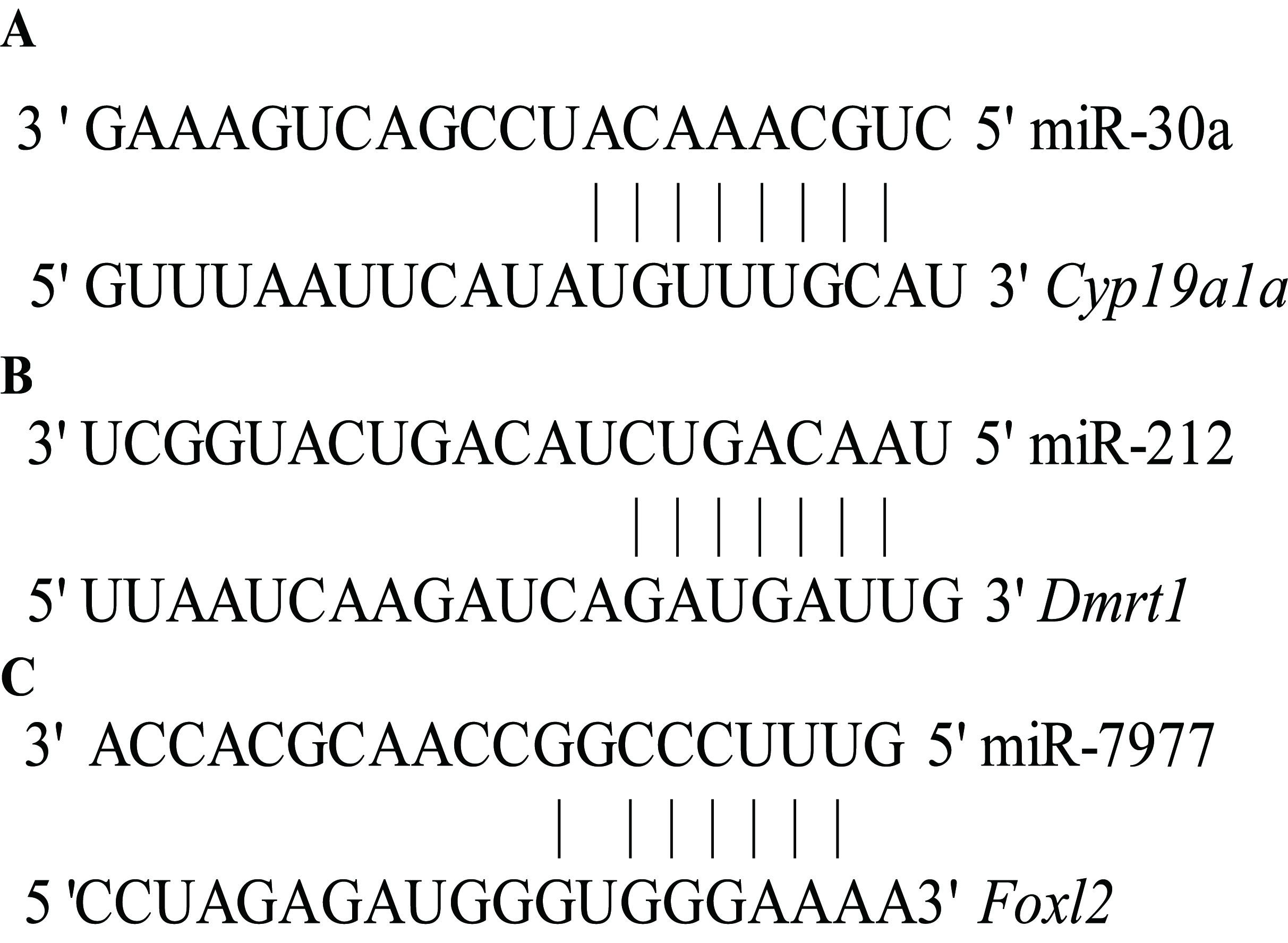

Supplement: Additional file 10: Figure S5. — Target prediction for selected miRNA expressed in ovaries and testis in tilapia. (A) Cyp19a1a, (B) Dmrt1, and (C) Foxl2 are predicted as potential targets of miR-30a, miR-212 and miR-7977, respectively. (TIF 1109 kb) [file 12864_2016_2636_MOESM10_ESM.tif]

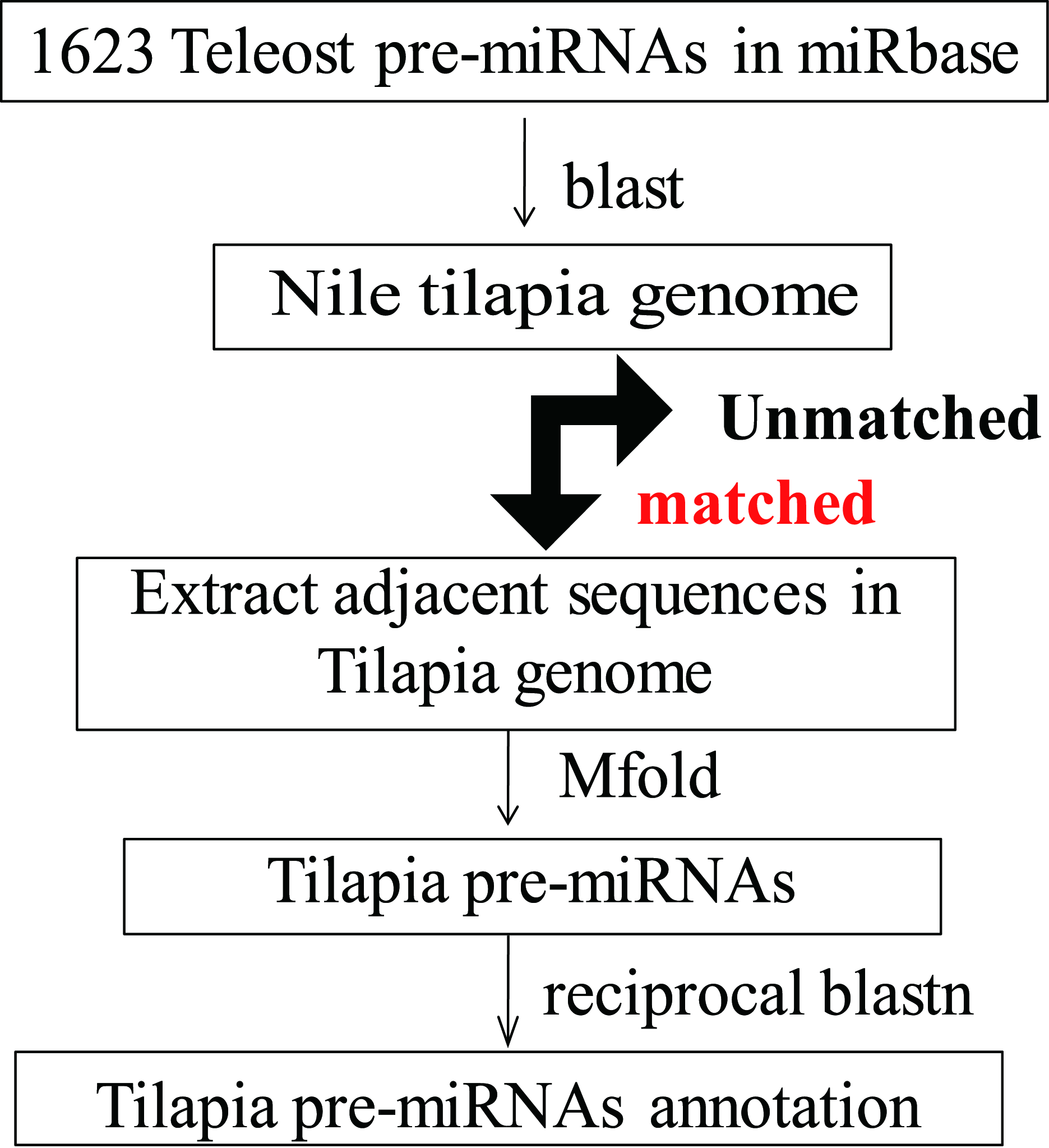

Supplement: Additional file 12: Figure S6. — Flowchart depicting the workflow for pre-miRNA prediction. A blastn similarity search of these pre-miRNAs against the tilapia genomic sequences [47] with an E-value cutoff of 0.001. The blastn hits were then manually inspected and compared with their query sequences in order to extract adjacent nucleotides (~30 bp) in both directions that might form part of the pre-miRNA. RNA secondary structure of the cichlid putative miRNA sequences was predicted using Mfold [75] to ensure proper stem–loop folding. A reciprocal blastn of the tilapia pre-miRNAs against known teleost pre-miRNAs was performed to identify the tilapia miRNA and to assign orthology. (TIF 1123 kb) [file 12864_2016_2636_MOESM12_ESM.tif]

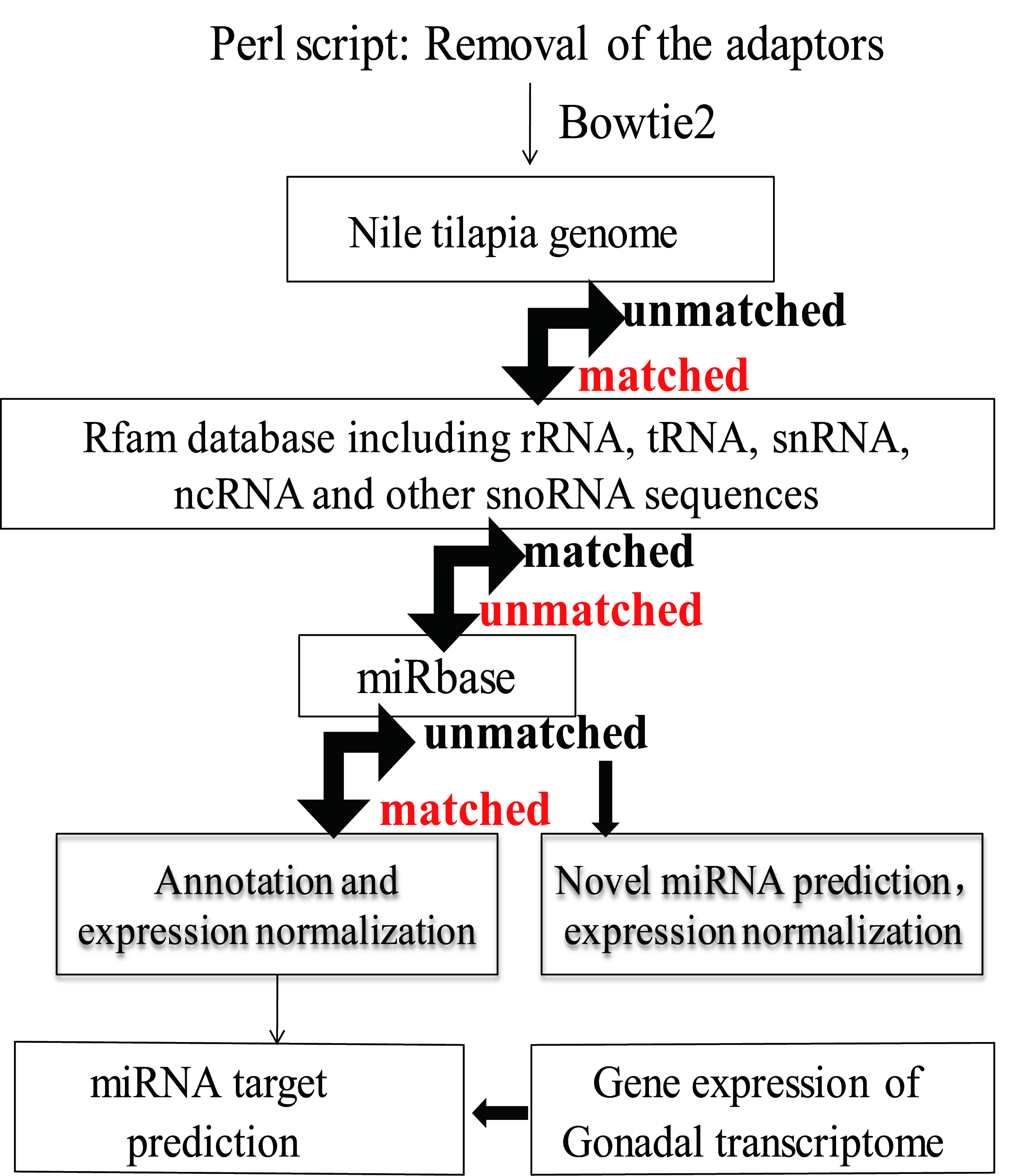

Supplement: Additional file 13: Figure S7. — Flowchart depicting the workflow for miRNA profiling, annotation and novel miRNA prediction. The workflow comprises of five parts namely: filter of the reads, quantification of known miRNAs, the novel miRNA prediction pipeline, target prediction, quantification of mRNA. The quantification of known miRNAs and prediction of novel miRNAs was done using Mfold, and the quantification of mRNAs was performed by Tophat2 [81] and Cufflinks [82]. (TIF 1823 kb) [file 12864_2016_2636_MOESM13_ESM.tif]
